# Identification and functional analysis of CCN6 variants in progressive pseudorheumatoid dysplasia: Exploring the potential role of ferroptosis and apoptosis in chondrocytes

**DOI:** 10.1016/j.gendis.2025.101564

**Published:** 2025-02-20

**Authors:** Yueyang Sheng, Shan Li, Ying Wang, XinYu Wang, Yanzhuo Zhang, Chengai Wu, Xu Jiang

**Affiliations:** a Department of Molecular Orthopaedics, National Center for Orthopaedics, Beijing Research Institute of Traumatology and Orthopaedics, Beijing Jishuitan Hospital, Capital Medical University, Beijing 100032, China; b Department of Orthopaedics, National Center for Orthopaedics, Beijing Jishuitan Hospital, Capital Medical University, Beijing 100032, China

Progressive pseudorheumatoid dysplasia (PPD, MIM 603400) is a rare autosomal recessive skeletal disorder that profoundly impairs joint function and diminishes quality of life. It is characterized by disproportionate short stature, extensive cartilage damage, and progressive joint enlargement symptoms typically including joint pain, stiffness, and swelling, initially affecting the interphalangeal joints before progressively involving larger joints and the spine.^1^ This progression often leads to severe joint contractures, spinal deformities, and gait abnormalities, significantly restricting mobility and overall well-being. The complexity of these complications highlights the critical role of genetic analysis in achieving an accurate diagnosis.^2^

PPD has been linked to variations in the *CCN6* gene, located on chromosome 6q22. This gene plays a crucial role as it encodes a growth factor protein abundantly found in connective tissue, particularly in synoviocytes and chondrocytes.^3^ *CCN6*, also known as WISP3 (Wnt-induced secreted protein 3), is essential for the development and maintenance of bone and cartilage. It facilitates these processes by regulating the synthesis of type II collagen and aggrecan—key components required to maintain cartilage homeostasis.^3^

We report a non-consanguineous patient presenting with PPD, characterized by narrowed joint spaces, reduced bone density, joint swelling, restricted joint mobility, and vertebral morphological abnormalities (Fig. 1A). A comprehensive summary of the patient's clinical symptoms is provided in Table S1. An analysis of the inheritance pattern identified potential pathogenic variants in the proband (Table S2). Through bioinformatics methods, two pathogenic variants in the *CCN6* gene were identified and subsequently confirmed by genomic DNA amplification using CCN6-E2-F/R primers (Table S3). Whole-exome sequencing and additional bioinformatics analysis revealed heterozygous variants, c.624dup (p.Cys209MetfsTer21) and c.136C > T (p.Gln46Ter), in the *CCN6* gene (Fig. 1C). Detailed quality control metrics for the whole-exome sequencing are outlined in Table S4. The maternal origin of the c.624dup (p.Cys209MetfsTer21) variant was confirmed, while the paternal transmission of the c.136C > T (p.Gln46Ter) variant was inferred but could not be definitively validated due to the absence of peripheral blood samples from the father. The pathogenicity of the c.624dup and c.136C>T mutations were established using cross-species conservation data (Fig. 1D), bioinformatics evaluations, and co-segregation analyses. According to the American College of Medical Genetics and Genomics (ACMG) guidelines, this mutation is classified as “pathogenic”. These findings provide a molecular basis for the PPD phenotype observed in our patient.

**Figure 1:**
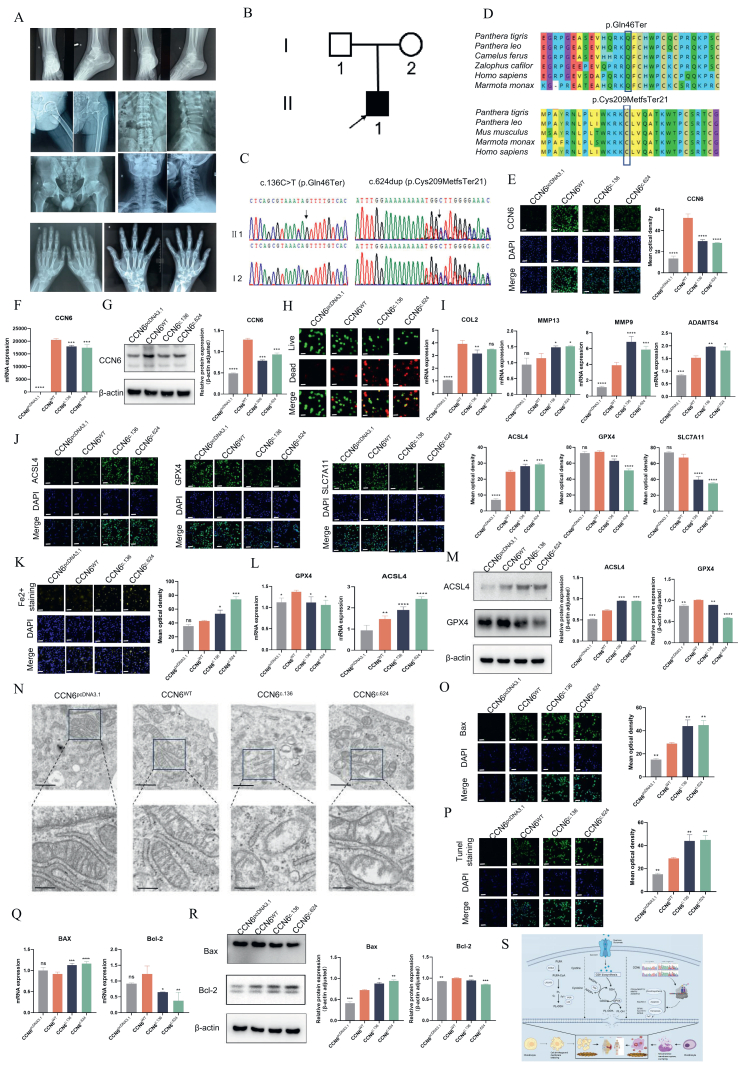
Identification and functional analysis of CCN6 variants in progressive pseudorheumatoid dysplasia. **(A)** Radiographic images showed progressive joint deterioration over time in patient II1. The series illustrates the gradual degeneration observed in the interphalangeal, hip, vertebral, and ankle joints. **(B)** Familial association and disease severity indicators. The black arrows mark the proband, while the open and filled circles and squares denote unaffected and affected individuals, respectively, highlighting the familial transmission and impact of the disease. **(C)** Sequence chromatograms of CCN6 mutations from Sanger sequencing. The chromatograms illustrate compound heterozygous mutations, c.136C>T (p.Gln46Ter) and c.624dup (p.Cys209MetfsTer21), identified in the patient. The mutation c.624dup was detected in his mother, appearing in a heterozygous state. **(D)** Comparative conservation analysis of CCN6 residues. A detailed comparison of p.Gln46 and p.Cys209 residues across multiple species underscores their evolutionary conservation. **(E)** Subcellular localization of CCN6 variants. The subcellular localization of CCN6^WT，^CCN6^c.136^ and CCN6^c.624^ was analyzed in transfected cells using an anti-CCN6 antibody. DAPI staining (blue) was employed to visualize the nuclei. Scale bar = 100 μm. **(F)** mRNA expression analysis in human primary chondrocytes. Gene expression alterations were evaluated following transfection with various CCN6 plasmids. **(G)** Protein levels and quantitative analysis of CCN6. The evaluation was conducted by assessing CCN6 and a corresponding loading control. **(H)** Live/dead staining of transfected human primary chondrocytes. A comparative viability assessment was performed on human primary chondrocytes expressing CCN6^WT^, CCN6^c.136^, and CCN6^c.624^. Control cells were transfected using pcDNA3.1. Scale bar = 100 μm. **(I)** mRNA expression of ECM-degrading enzymes and COLII. An analysis was conducted on human primary chondrocytes transfected with various plasmids to examine their impact on extracellular matrix composition. **(J)** Immunofluorescent imaging of ACSL4, GPX4 and SLC7A11 protein expressions. Quantitative analysis was conducted to assess expression levels. Scale bar = 100 μm. **(K)** Fe^2+^ expression analysis via immunofluorescence. Quantitative analysis was performed on human primary chondrocytes transfected with various CCN6 constructs, including control cells transfected with pcDNA3.1. Scale bar = 100 μm. **(L)** mRNA expression of ferroptosis-related genes. The expression levels of ferroptosis-related genes were evaluated in cells transfected with various CCN6 constructs to investigate the molecular pathways potentially involved in cell death. **(M)** Protein levels and quantification of ferroptosis markers. The levels of ACSL4 and GPX4 were analyzed using CCN6^c.136^ and CN66^c.624^. **(N)** Ultrastructural analysis via transmission electron microscopy. Transmission electron microscopy images of human primary chondrocytes transfected with various plasmids were analyzed to evaluate cellular morphology. Scale bar = 2 or 6 μm. **(O)** Quantitative analysis of Bax protein expression and representative immunofluorescent images. Scale bar = 100 μm. **(P)** TUNEL staining for apoptotic cells. **(Q)** Expression analysis of Bax and Bcl-2 mRNA in human primary chondrocytes transfected with distinct CCN6 constructs. **(R)** Quantitative analysis of Bax and Bcl-2 protein levels, with β-actin used as a normalization reference. **(S)** Schematic overview of ferroptosis and apoptosis mechanisms. Molecular pathways triggered in chondrocytes by CCN6 mutations were illustrated.

To date, the Human Genetic Mutation Database (HGMD) has documented 86 mutations within the *CCN6* gene. These encompass 37 missense or nonsense mutations (43%), 9 splice mutations, 24 small deletions, and 12 small insertions, as outlined in Table S5. Notably, variants such as c.136C > T (p.Gln46Ter) and c.624dup (p.Cys209MetfsTer21) have been previously associated with PPD. Among cases involving the c.136C > T mutation, all affected individuals displayed typical phenotypic features, with clinical and imaging findings aligning with PPD characteristics.

To explore the functional implications of *CCN6* mutations, we constructed expression plasmids encoding both the wild-type and mutant forms of the *CCN6* gene and transfected them into primary human chondrocytes. The effects of these mutations on CCN6 protein expression, subcellular localization, and chondrocyte viability were meticulously evaluated using techniques including immunofluorescence staining (Fig. 1E), quantitative real-time PCR (Fig. 1F), and Western blot analysis (Fig. 1G). This study aims to unravel the intricate pathogenic mechanisms underlying PPD caused by *CCN6* mutations, with a particular emphasis on the mutants' capacity to induce ferroptosis and apoptosis in chondrocytes.

Significant differences in expression were observed between cells transfected solely with the vector, which exhibited minimal immunofluorescence staining, and those transfected with wild-type *CCN6*, where fluorescence signals were clearly present in both the cytoplasm and nucleus. In contrast, cells expressing the CCN6^c.136^ and CCN6^c.624^ variants displayed significantly reduced immunofluorescence signals, which were predominantly localized within the cytoplasm (Fig. 1E). Furthermore, the mRNA and protein levels of CCN6 in chondrocytes transfected with these mutant variants were notably lower than those in cells transfected with the wild-type gene, consistent with the immunofluorescence results (Fig. 1F, G).

Live/dead staining was utilized to evaluate the impact of these variants on cell viability. In this assay, live cells exhibited green fluorescence, while dead cells displayed red fluorescence. Compared with the group transfected with CCN6^WT^, the groups expressing CCN6^c.136^ and CCN6^c.624^ showed a marked reduction in viable cells, indicating significant inhibition of chondrocyte proliferation (Fig. 1H). Furthermore, quantitative real-time PCR analysis demonstrated a considerable increase in the relative expression levels of ADAMTS4, MMP13, and MMP9 in chondrocytes carrying the CCN6^c.136^ and CCN6^c.624^ mutations when compared with those transfected with CCN6^WT^. In contrast, the mRNA expression level of COLII was significantly decreased in chondrocytes harboring the CCN6^c.136^ mutation relative to CCN6^WT^ (Fig. 1I). These results indicate that these two variants profoundly affect cell viability, proliferation, and the synthetic as well as metabolic pathways governing both synthesis and degradation processes within chondrocytes.

While various forms of chondrocyte cell death have been linked to cartilage-related disorders, this study specifically examines ferroptosis and apoptosis due to their well-documented roles in maintaining cartilage homeostasis and contributing to disease progression. Apoptosis has been extensively associated with chondrocyte aging and the breakdown of the extracellular matrix, whereas ferroptosis, though a more recently identified mechanism, has gained increasing recognition for its contribution to cartilage degeneration in the presence of oxidative stress and metabolic imbalances.

The immunofluorescence analysis of the ferroptosis marker ACSL4 showed elevated expression levels in chondrocyte groups transfected with CCN6^c.136^ and CCN6^c.624^ compared with those transfected with CCN6^WT^ (Fig. 1J). Conversely, the fluorescence intensity of GPX4 and SLC7A11 was reduced in chondrocytes harboring the CCN6^c.136^ and CCN6^c.624^ mutations, relative to those expressing CCN6^WT^ (Fig. 1J). This distinct expression pattern underscores a potential mechanistic link between these *CCN6* mutations and the induction of ferroptosis in chondrocytes.

Using the FerroOrange dye, we detected a pronounced increase in Fe^2+^ levels within chondrocytes carrying the CCN6^c.136^ and CCN6^c.624^ mutations, indicating elevated ferroptosis (Fig. 1K). Consistent with these observations, quantitative real-time PCR and Western blot analyses demonstrated a significant reduction in the expression levels of GPX4 and SLC7A11 in the mutant cells compared with the wild-type, while ACSL4 expression was notably up-regulated, aligning with the immunofluorescence results (Fig. 1L, M). Furthermore, transmission electron microscopy revealed mitochondrial fragmentation, reduced cristae, and increased membrane density in the mutant cells (Fig. 1N). Taken together, these findings suggest that mutations in the *CCN6* gene disrupt cellular homeostasis in chondrocytes by promoting ferroptosis, which may contribute to the pathological mechanisms underlying conditions such as PPD.

The immunofluorescence analysis of the apoptosis marker Bax revealed heightened expression in chondrocyte groups harboring CCN6^c.136^ and CCN6^c.624^ mutations, compared to CCN6^WT^ (Fig. 1O). TUNEL staining further substantiated that these mutations significantly elevated apoptosis in chondrocytes (Fig. 1P). Complementary results from quantitative real-time PCR (Fig. 1Q) and Western blot analyses (Fig. 1R) demonstrated a pronounced up-regulation of Bax and a corresponding down-regulation of Bcl-2 in the mutant cells, aligning with the immunofluorescence findings. Collectively, these results indicate that *CCN6* mutations not only undermine chondrocyte viability but also actively promote cell death via two distinct mechanisms: ferroptosis and apoptosis. The emphasis on these pathways emerged from the identification of specific molecular markers and signaling alterations in chondrocytes carrying CCN6 mutations, offering clear evidence of their role in the cellular response to these genetic changes.

The significance of the CCN6^c.136^ and CCN6^c.624^ mutations in activating these death pathways sheds critical light on the pathological mechanisms underlying PPD. This study reveals that mutations in the CCN6 gene contribute to PPD by disrupting chondrocyte homeostasis, leading to the induction of ferroptosis and apoptosis. These processes accelerate chondrocyte degradation while suppressing synthesis, resulting in molecular disturbances that severely impair the functionality and survival of chondrocytes, thereby driving the progression of PPD. Consequently, this research provides valuable insights into the pathological basis of PPD associated with CCN6 mutations and emphasizes the need for further investigation to confirm and expand these findings (Fig. S1). Notably, future studies should also focus on exploring other cell death mechanisms, such as necrosis and autophagy, within the context of CCN6-related pathologies.

## CRediT authorship contribution statement

**Yueyang Sheng:** Writing – original draft, Methodology, Investigation, Data curation, Conceptualization. **Shan Li:** Writing – original draft, Project administration, Methodology, Investigation, Data curation, Conceptualization. **Ying Wang:** Methodology, Data curation. **XinYu Wang:** Methodology, Data curation. **Yanzhuo Zhang:** Data curation. **Chengai Wu:** Writing – review & editing, Project administration, Investigation, Conceptualization. **Xu Jiang:** Writing – review & editing, Project administration, Investigation, Conceptualization.

## Ethics declaration

All procedures were conducted in strict compliance with the ethical standards set forth by the institutional and national research committees, as well as the 1964 Helsinki Declaration and its subsequent amendments or equivalent ethical guidelines. The study was reviewed and approved by the Ethics Committee of Beijing Jishuitan Hospital, Capital Medical University (Approval Code: 201611-03). Informed consent was obtained from all participating patients prior to their inclusion in the study.

## Funding

This research was supported by the Beijing Natural Science Foundation (China) (No. 7244286), the 10.13039/501100005088Beijing Municipal Health Commission (China) (No. BJRITO-RDP-2025), and the Beijing Natural Science Foundation-Haidian Original Innovation Joint Fund (China) (No. L222089).

## Conflict of interests

The authors declared no conflict of interests.
