# Supplementary material for: Identification and functional analysis of CCN6 variants in progressive pseudorheumatoid dysplasia: Exploring the potential role of ferroptosis and apoptosis in chondrocytes
Source: Genes Dis. 2025 Feb 20;13(1):101564. doi: 10.1016/j.gendis.2025.101564 (PMC12624680; doi:10.1016/j.gendis.2025.101564)
Supplement: Multimedia component 1 [file mmc1.docx]

**MATERIALS AND METHODS**

**Collecting Blood Samples**

Upon receiving informed, written consent pertaining to genetic analysis and data publication from two participants, the study was carried out adhering to the Helsinki Declaration and was granted approval by the Ethics Committee of Beijing Jishuitan Hospital, affiliated with Capital Medical University. Peripheral blood samples (5 ml) were retrieved in EDTA tubes. Genomic DNA was extracted with the QIAamp DNA Blood Midi Kit (Qiagen, Hilden, Germany) and quantified using the Nanodrop 2000 spectrophotometer (Thermo Scientific).

**Whole Exome Sequencing (WES) and Data Analysis**

Exome sequencing of the proband (II-1) was conducted using the Agilent SureSelect Human All Exon V6 kit (Agilent Technologies, USA). Sequencing libraries were prepared and sequenced on the Illumina HiSeq 2000 platform (Illumina, San Diego, CA). Initial raw data composed of fluorescence image files were subjected to base calling to generate short reads. Adapter sequences and low-quality reads were filtered out, yielding high-quality clean data. These clean reads were mapped to the human reference genome sequence (GRCh37/hg19) using Burrows-Wheeler Aligner software. Quality control metrics for the whole-exome sequencing are detailed in Table S1.

**Conservation analysis**

Amino acid sequences of CCN6 were sourced from the NCBI Protein database in FASTA format. Multiple sequence alignments were performed using MEGA software, which facilitated comparison of sequences across various species.

**construction and Transfection of Plasmids**

Wild-type of pcDNA3.1(+)-CN66 plasmids were procured from Nuosai (Beijing, China), and mutant expression vectors—Gln46Ter and Cys209MetfsTer21—were generated using pcDNA3.1-CN66 as the template. These were created using PCR with mutagenic primers and verified via Sanger sequencing. Human primary chondrocytes (Pricella, China) were cultured in DMEM enriched with 10% FBS, L-glutamine, and 1% penicillin-streptomycin in a 37 °C, 5% CO2 environment. Transfection was carried out using Lipofectamine 3000 (Invitrogen, USA) to introduce wild-type and mutant CCN6 genes (c.136, c.624), along with the pcDNA3.1(+) plasmid as a control. These transfected cells were designated as WT (wild-type), MUT (mutant), and Control, respectively.

**Live/Dead staining**

After 24 hours of transfection, live/dead staining solution (500 μl) was introduced to the 12-well plates, then incubated at 37°C in darkness for half an hour. Live cells displayed green fluorescence, whereas dead cells showed red under a fluorescencent microscope. Fluorescent images of live/dead cells were documented using a fluorescence microscope (Molecular Imaging Devices, America) and analyzed with Image J software.

**Fenton reaction**

The Fenton reaction primarily involves the catalysis of hydrogen peroxide (H2O2) via ferrous ions (Fe^2+^). This produces toxic hydroxyl radicals (OH) and initiates lipid peroxidation in cells. FerroOrange, a unique fluorescent probe, enables efficient, quick, and straightforward fluorescence imaging of intracellular Fe^2+^ in live cells. After 24 hours of transfection at 37°C in 5% CO2, the culture medium was replaced. A working solution of FerroOrange was added at a concentration of 1 μmol/L, then incubated for 30 minutes at 37°C with 5% CO2. According to this protocol (FerroOrange, Dojindo), cells were analyzed using a fluorescence microscope (Molecular Imaging Devices, America).

**Transmission Eelectron Microscopy (TEM)**

Samples were fixed employing an electron microscope fixative (Servicebio, G1102) and subjected to dehydrated at room temperature. Following this, the dehydrated samples were osmotically embedded using acetone and an 812 embedding agent. Ultrathin sections (60-80 nm) were sliced from the embedded samples utilizing an ultrathin sectioning machine and placed onto copper grids. The sections were subjected to staining with uranyl acetate and lead citrate to improve contrast. Images were captured using a digital camera coupled with the Transmission Electron Microscope (HITACHI, HT7800/HT7700).

**Immunofluorescence Staining**

48 hours post-transfection, cells were fixed using 4% paraformaldehyde for 15 minutes at room temperature. The cells were permeabilized with 0.2% Triton X-100 in PBS for 20 minutes to facilitate antibody penetration. Following permeabilization, the cells were blocked with 1% BSA in PBS for one hour at room temperature to reduce non-specific binding. For immunostaining, the cells were incubated with various primary monoclonal antibodies: CCN6 (Abcam-ab103263), SLC7A11 (proteintech-26864-1-AP), GPX4 (proteintech-67763-1-Ig), ACSL4 (Abcam-ab205199), Bax (CST-2772T), and Bcl-2 (Abcam-ab692) at 37°C for 2 hours. Afterward, the cells were washed thrice with PBS to remove any unbound antibodies. The cells were subsequently incubated with anti-rabbit IgG secondary antibodies (Abcam-ab150077, diluted 1:1000 in the blocking solution) for one hour at room temperature. Following another PBS wash, the cells were stained with DAPI nuclear staining solution for 5 minutes to visualize nuclei. Excess DAPI stain was removed with PBS to ensure distinct nuclear staining. The immunostained cells were then imaged using a content screening system (Molecular Devices, America).

**Quantitative real-Time PCR**

Total RNA was extracted from chondrocytes using the MiniBEST Universal RNA Extraction Kit (TaKaRa, China), and the synthesis of cDNA was performed with the PrimeScript™ RT reagent Kit with gDNA Eraser (perfect real-time) (TaKaRa, China). Quantitative real-time PCR was conducted using either the Real-Time PCR System or the ABI 7900HT Sequence Detection System (ABI, America). The expression levels of relevant genes in blood samples were assessed utilizing the SYBR Green approach,with GAPDH operating as the normalization control. The sequences of the gene primers are listed in Table S2.

**Western Blotting Analysis**

Protein samples were procured using a total protein extraction kit (Keygen Biotech, China), and upon centrifugation to eliminate leftover debris, the protein concentration was verified via the Bradford method. The samples were then mounted onto SDS-PAGE gels before their transfer to PVDF membranes. The membranes were blocked for one hour and overnight at 4°C with primary antibodies (1:1000 dilution) targeting the CCN6 (Proteintech-26406-1-AP), GPX4 (Proteintech-67763-1-Ig), ACSL4 (Abcam-ab205199), Bax (CST-2772T), Bcl-2 (Abcam-ab692), and β-actin (Abcam-ab8226) proteins. After washing, the membranes were incubated with horseradish peroxidase-conjugated secondary antibodies at 4°C for 4 hours. Immunoreactivity was visualized using an enhanced chemiluminescence substrate (Sage Creation), and protein expression levels were quantified using Image J software.

**Statistical analysis**

The data are represented as mean ± standard deviation and were scrutinized using GraphPad Prism 9.0. Differences among multiple groups were evaluated using one-way analysis of variance (ANOVA), and P values less than 0.05 were considered statistically significant.
