# Supplementary material for: Identification and functional analysis of CCN6 variants in progressive pseudorheumatoid dysplasia: Exploring the potential role of ferroptosis and apoptosis in chondrocytes
Source: Genes Dis. 2025 Feb 20;13(1):101564. doi: 10.1016/j.gendis.2025.101564 (PMC12624680; doi:10.1016/j.gendis.2025.101564)
Supplement: Multimedia component 2 [file mmc2.docx]

| clinical symptoms | Detailed information |
| --- | --- |
| Characteristic | II 1 |
| Gender | Male |
| Family history | - |
| Age (years) | 16 |
| Age at onset (years) | 10 |
| Site of pathological changes | Spine, hip, interphalangeal and wrist joints |
| Height (cm) | 163 |
| Arthralgia | + |
| Spinal nerve involvement | - |
| Enlargement and stiffness of joint | + |
| Activity limitation | + |
| Spine malformation | + |
| Narrowing of intervertebral and joint space | + |
| Vertebral deformities | + |
| Acetabular fossa deformation | + |
| Narrowing of the interphalangeal joints | + |

Table 1 Summary of clinical symptoms of the proband.
