# Supplementary material for: Identification and functional analysis of CCN6 variants in progressive pseudorheumatoid dysplasia: Exploring the potential role of ferroptosis and apoptosis in chondrocytes
Source: Genes Dis. 2025 Feb 20;13(1):101564. doi: 10.1016/j.gendis.2025.101564 (PMC12624680; doi:10.1016/j.gendis.2025.101564)
Supplement: Multimedia component 3 [file mmc3.docx]

Table S2 Candidate pathogenic variants in the proband.

| CCN6 | c.624dup(p.Cys209MetfsTer21) | c.136C>T (p.Gln46Ter) |
| --- | --- | --- |
| mutation ID | 6:112389433:-/A | 6:112382281:C/T |
| hg38 | 6:112068230:-/A | 6:112061078:C/T |
| RS number | rs782739258 |  |
| gene name | CCN6 | CCN6 |
| Variant score | 0.806998400238781 | 0.8214365 |
| Gene score | 0.576851156451234 | 0.576851156451234 |
| Causative score | 0.682288766162859 | 0.688365161070964 |
| phenotypic match | 0.73 | 0.73 |
| HGVS | NM_003880.4:c.624dup (p.Cys209MetfsTer21)  NM_198239.2:c.624dup (p.Cys209MetfsTer21)  NR_125353.2:n.942dup  NR_125354.3:n.769dup | NM_003880.4:c.136C>T (p.Gln46Ter) NM_198239.2:c.136C>T (p.Gln46Ter) NR_125353.2:n.390C>T NR_125354.3:n.217C>T |
| Type of mutation | frameshift_variant | stop_gained |
| sequencing depth | 102 | 189 |
| Allele depth | 66,36 | 88,101 |
| genotype (genetics) | HET (0/1) | HET (0/1) |
| Genotype quality value | 99 | 99 |
| Genotypic credibility | High | High |
| Maximum group frequency | 0.000524 | - |
| ExAC | 0.000381 | - |
| ExAC EAS | 0.000524 | - |
| 1KG | - | - |
| 1KG EAS | - | - |
| gnomAD exome | 0.00027 | - |
| gnomAD exome EAS | 0.000203 | - |
| gnomAD genome | - | - |
| gnomAD genome EAS | - | - |
| MDD | - | - |
| harmful | High | High |
| CADD |  |  |
| GERP++ |  | 1.06 |
| CROG | PAT | PAT |
| ClinVar |  |  |
| HGMD | PAT | PAT |
| ACMG evidence | PM2 | PM2 |
| ACMG classifications | VUS | VUS |
| clinical significance | PAT | PAT |
| Mutation Tags | HRun=true |  |
| PubMed | [20426955](https://www.ncbi.nlm.nih.gov/pubmed/20426955) | [19064006](https://www.ncbi.nlm.nih.gov/pubmed/19064006) |
