# Supplementary material for: Identification and functional analysis of CCN6 variants in progressive pseudorheumatoid dysplasia: Exploring the potential role of ferroptosis and apoptosis in chondrocytes
Source: Genes Dis. 2025 Feb 20;13(1):101564. doi: 10.1016/j.gendis.2025.101564 (PMC12624680; doi:10.1016/j.gendis.2025.101564)
Supplement: Multimedia component 4 [file mmc4.docx]

Table S3. Sequences of primers used in qRT-PCR.

| Genes |  | Sequences |
| --- | --- | --- |
| WISP3  GPX4  SLC7A11  BaX  Bcl-2  GAPDH | Forward  Reverse  Forward  Reverse  Forward  Reverse  Forward  Reverse  Forward  Reverse  Forward  Reverse | ACTGTAGCCTGGAACCATTACT  TGGTCACCCTGTTAGATATTCCC  GAGGCAAGACCGAAGTAAACTAC  CCGAACTGGTTACACGGGAA  GGTCCATTACCAGCTTTTGTACG  AATGTAGCGTCCAAATGCCAG  GAGGAGGAGATCGTGTTTCCA CCAGCTCTAGTAGCAGCGTC  GTCTTCGCTGCGGAGATCAT  CATTCCGATATACGCTGGGAC  TGTGGGCATCAATGGATTTGG  ACACCATGTATTCCGGGTCAAT |
