# Supplementary material for: Identification and functional analysis of CCN6 variants in progressive pseudorheumatoid dysplasia: Exploring the potential role of ferroptosis and apoptosis in chondrocytes
Source: Genes Dis. 2025 Feb 20;13(1):101564. doi: 10.1016/j.gendis.2025.101564 (PMC12624680; doi:10.1016/j.gendis.2025.101564)
Supplement: Multimedia component 5 [file mmc5.docx]

Table S4 Sequencing data statistics

| Capture kits | Agilent SureSelect Human All Exon V6 |
| --- | --- |
| Target area size | 60.46M |
| Raw data volume | 12.39G |
| Amount of data filtered | 12.36G |
| Genome matching rate | 99.86% |
| The ratio of duplicate reads | 19.37% |
| Unique comparison rate | 98.86% |
| mismatch ratio | 0.3340% |
| On target rate | 54.55% |
| Average sequencing depth of target region | 111.35 |
| 1X coverage of target area | 99.78% |
| 10X coverage of target area | 97.97% |
| 20X coverage of target area | 94.77% |
| 50X coverage of target area | 79.92% |
